# Hormone immunolabeling in resin-embedded *Arabidopsis* tissues

**DOI:** 10.1016/j.xpro.2023.102514

**Published:** 2023-08-14

**Authors:** Humberto Herrera-Ubaldo, Héctor-Rogelio Nájera-González, Valentín Luna-García, Nayelli Marsch-Martínez, Stefan de Folter

**Affiliations:** 1 Unidad de Genómica Avanzada (UGA-LANGEBIO), Centro de Investigación y de Estudios Avanzados del Instituto Politécnico Nacional (CINVESTAV-IPN), Irapuato, Guanajuato 36824, México; 2 Departamento de Biotecnología y Bioquímica, Centro de Investigación y de Estudios Avanzados del Instituto Politécnico Nacional (CINVESTAV-IPN), Irapuato, Guanajuato 36824, México

**Keywords:** Model Organisms, Plant Sciences, Molecular Biology, Antibody

## Abstract

Here, we present a protocol for immunolabeling of molecules in *Arabidopsis* tissues. We describe steps for tissue fixation and embedding in resin of microtome-derived sections, immunolabeling using fluorescent and non-fluorescent secondary antibodies, and visualization of cytokinin and auxin molecules. This protocol is suitable to study reproductive structures such as inflorescences, flowers, fruits, and tissue-culture-derived samples. This protocol is useful for studying the distribution of a wide range of molecules including hormones and cell wall components.

For complete details on the use and execution of this protocol, please refer to Herrera-Ubaldo et al. (2019).^1^

## Highlights

• Plant tissue fixation and embedding in resin to make thin tissue sections
• Immunolabeling to detect hormones in resin sections
• Immunolabeling using fluorescent and non-fluorescent secondary antibodies
• Visualization of cytokinin and auxin molecules in plant tissues

## Abstract (editor highlights)

Publisher’s note: Undertaking any experimental protocol requires adherence to local institutional guidelines for laboratory safety and ethics.

## Graphical abstract

**Figure:**
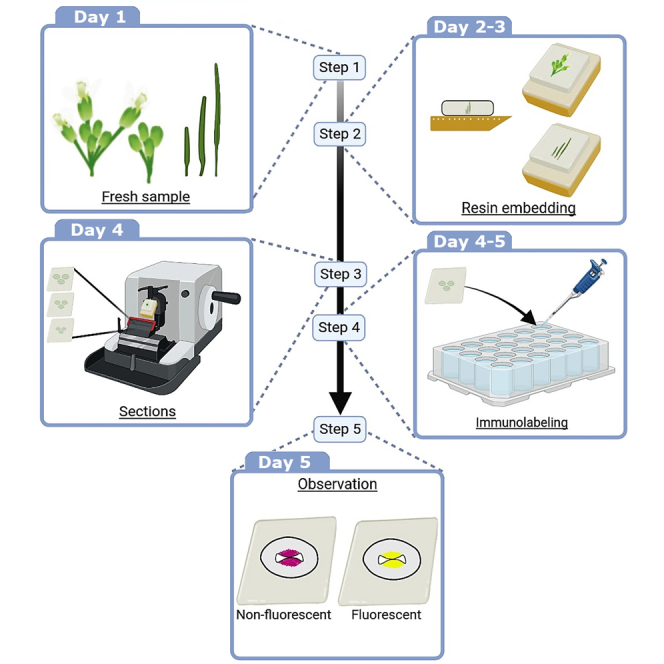

## Before you begin

**Figure 1:**
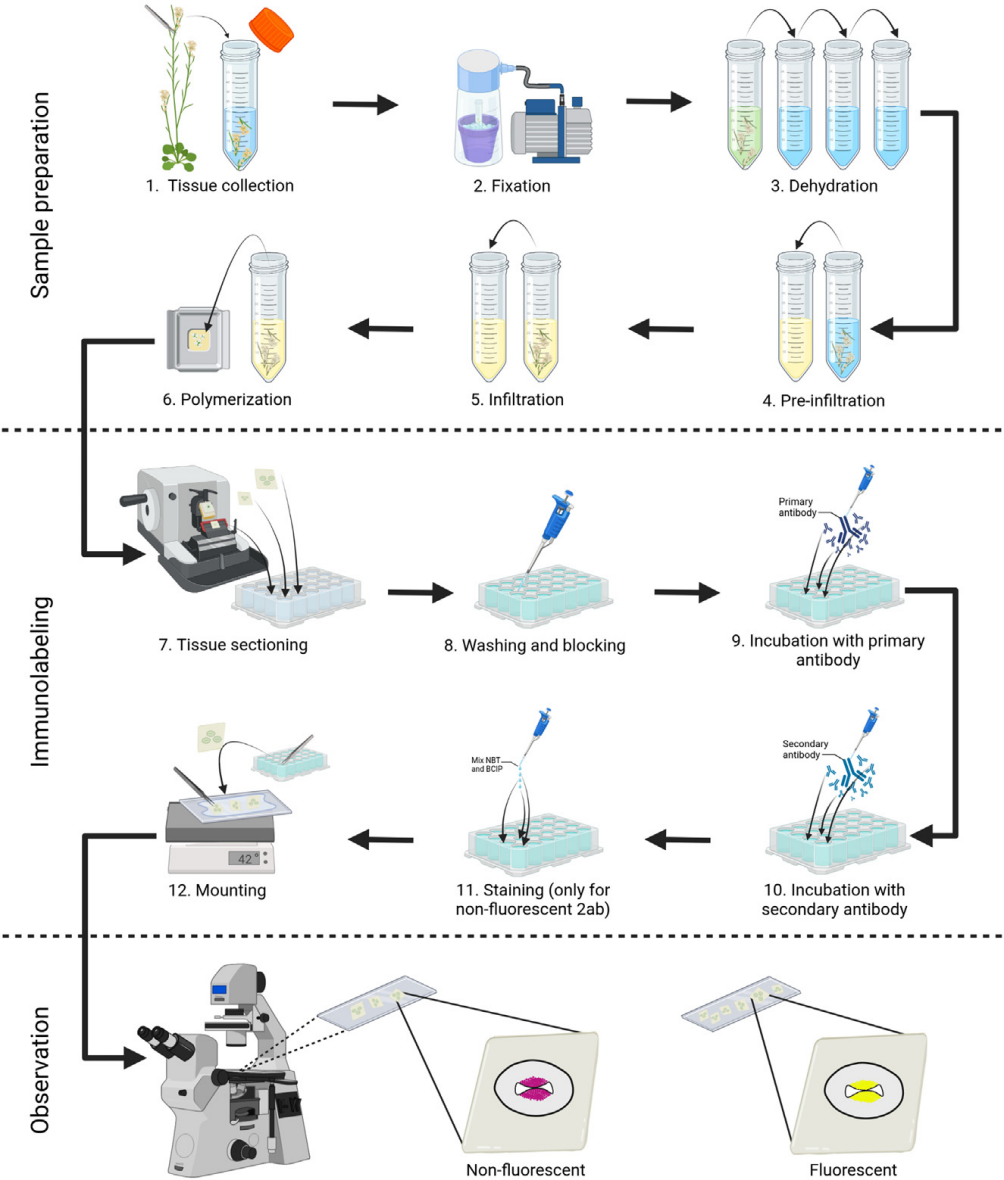
Overview of the immunolabeling protocol

**Figure 2:**
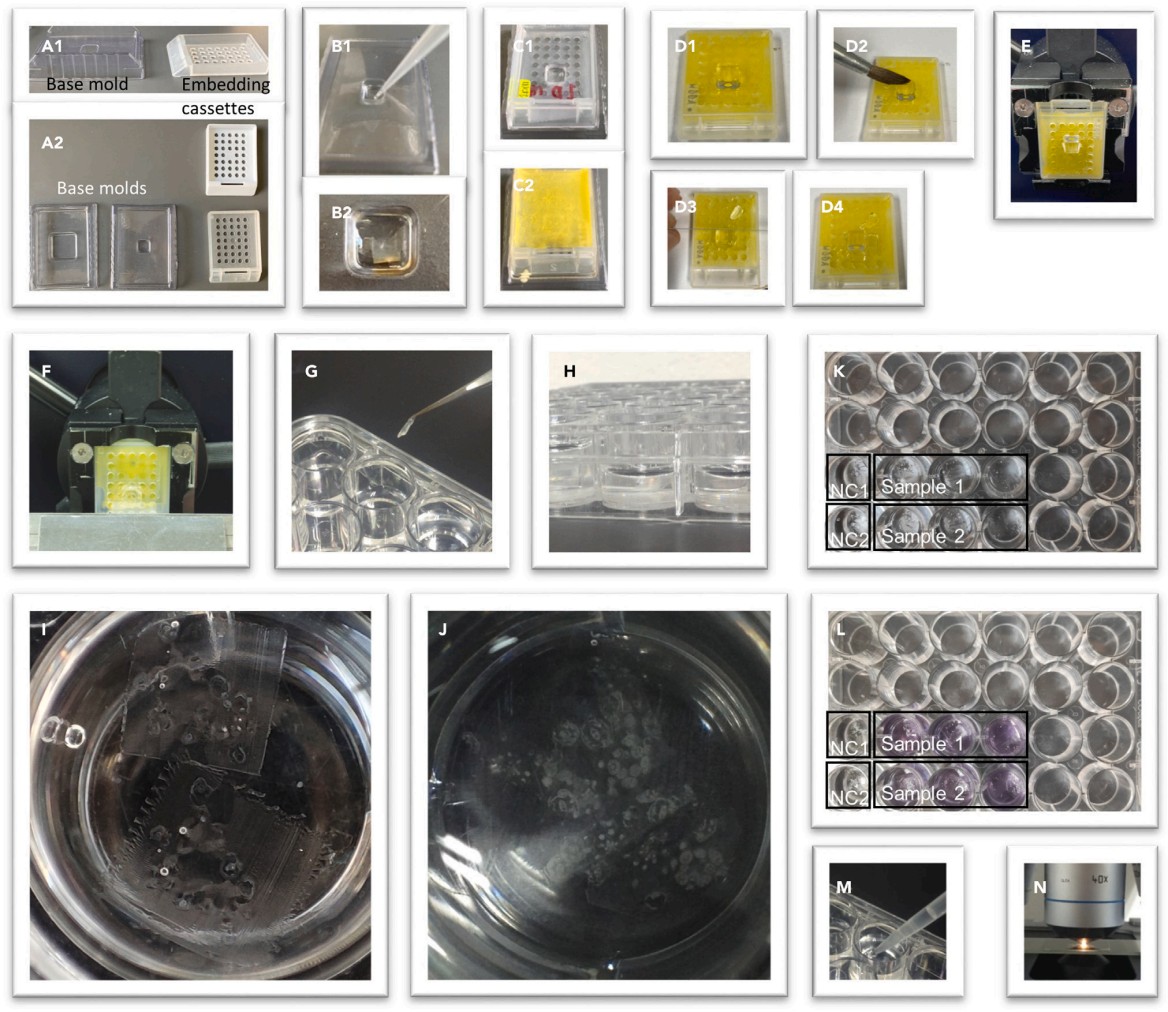
Details of how to use and handle a 24-well plate in the immunolabeling procedure (A) Examples of base molds and embedding cassettes (A1, A2). (B) Base mold, adding 200 μL Technovit polymerization solution (B1), followed by placing the samples and then cover them with more of the same solution (B2). (C) Embedding cassette placed over the base mold with the polymerized block (C1), followed by filling the cassette with the glue Technovit 3040 (yellow) (C2). (D) Trimming of the block, block can be wetted with water (D2) to easier trim the block with a knife (D3), final result of trimmed block (D4). (E) Cassette with glued block on it can be placed in the microtome. (F) Preparation of sections with a microtome. (G) Example of putting or removing a section from the 24-well plate. (H) Side view of the 24-well plate with solution. (I and J) Examples of sections floating on the solution in a well (top view). (K and L) Examples of a typical experiment using 8 wells: two for negative controls (NC1: no primary antibody: NC2: no secondary antibody) and 6 for samples (e.g., three wild-type and 3 mutant samples). In L) the color of the NBT-BCIP staining solution can be seen (when using an alkaline phosphatase-conjugated secondary antibody). (M) Example of how to do washing steps with a pipette. (N) Example of observing the samples under the microscope.

**Figure 3:**
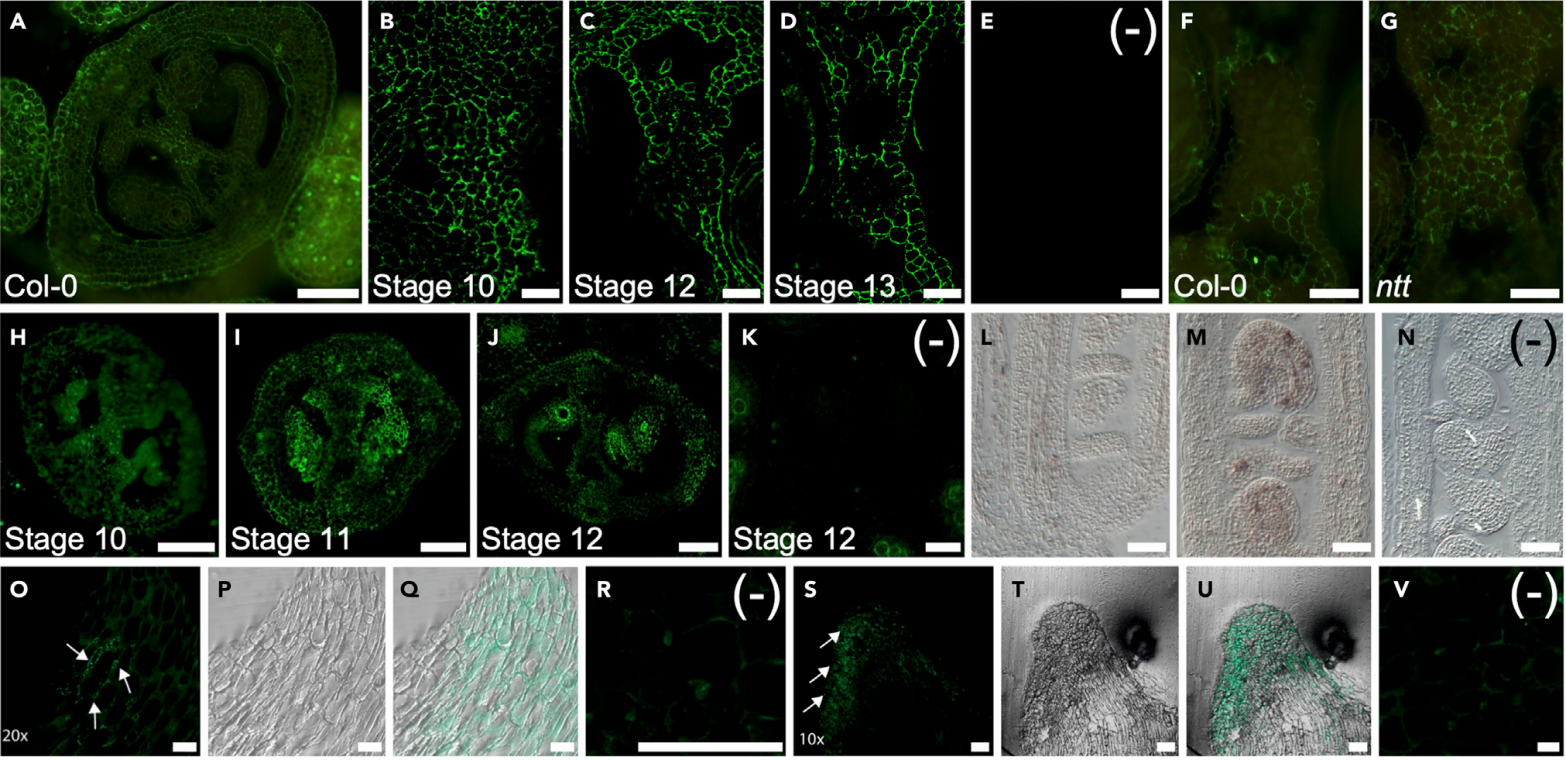
Immunolabeling of hormones and cell wall components (A) Mannan polysaccharide distribution in a transverse section of an Arabidopsis gynoecium. (B–D) Changes in mannan polysaccharide distribution during septum development in wild type Arabidopsis gynoecium.^2^. (F and G) Comparison of mannan polysaccharide distribution in the septum in the wild-type Col-0 and the *no transmitting tract* (*ntt*) mutant of stage 12 gynoecia in *Arabidopsis*.^1^. (H–J) Distribution of trans-zeatin during gynoecium development in Arabidopsis (unpublished, de Folter laboratory). (L and M) (L) Trans-zeatin (cytokinin) and (M) IAA (auxin) distribution in longitudinal section of an Arabidopsis gynoecium with visible ovules (unpublished, de Folter laboratory). (O–V) Trans-zeatin (O-Q) or IAA (S-U) immunolabeling in maize calli during *in vitro* plant regeneration.^3^ E,K,N,R, and V are the negative controls (no primary antibody). In A-K, O-V, a fluorescent DyLight 488 conjugated secondary antibody was used; in L-N, an AP-conjugated secondary antibody was used. Scale bars: A, H-N, O-R 50 μm, B-G 20 μm, S-V 100 μm.

Here, we describe how to perform immunolabeling of molecules in plant tissues embedded in resin (Figures 1 and 2). Depending on the antibody used, it is possible to visualize hormones such as auxin and cytokinin, or cell wall components such as mannans (Figure 3).^1^^,^^2^^,^^3^ This protocol is not limited, and can also be used for experiments that employ other antibodies or other plant tissues from Arabidopsis or other plant species. Furthermore, variations of this protocol can also be used to visualize microtubules using whole mount immunolocalization.^4^^,^^5^ Important, always investigate if an additional treatment must be performed after the fixation step to be able to detect specific molecules by immunolabeling. To detect hormones, no additional treatment is needed, however, to detect cell wall components such as mannans, an additional treatment is needed (See troubleshooting Section).^2^^,^^6^

### Sample embedding

**Timing: 3 days**

1. Tissue collection and fixation.
  a. Collect the sample in a tube containing cold fixation buffer.
    ***Note:*** Depending on the sample size you can use 2 mL or 50 mL tubes. A good volume ratio for solution:sample is 10:1. For small samples, the working volume could be 1 mL solution in 2 mL tubes, for larger samples (i.e., Arabidopsis inflorescences), 5 mL of solution in 50 mL falcon tubes should work. Consider collecting enough samples to have technical replicates and controls.
  b. Place the tube with the sample in a beaker with ice in a vacuum desiccator and apply vacuum (0.6 kPa) for 30 min.
    ***Note:*** The samples should sink as an indicator of good penetration of fixative. See materials and equipment Section for fixation buffer. When the aim is to observe hormones, the MTSB buffer must be included in the fixation buffer.
    ***Note:*** If the tissue does not sink after vacuum application, try to shake the tube to remove any air bubbles. If the samples do not sink, apply vacuum for 15 min more.
  c. Remove the tube from the vacuum chamber and place the tube on ice and incubate for 2.5 h.
  d. Remove fixation buffer and rinse 3 times with Milli-Q water (use the same volume as fixative used).
    ***Note:*** Plant tissue will still be flexible.
    **CRITICAL:** Fixation buffer is toxic due to the presence of paraformaldehyde, should be prepared in the fume hood. After use, discard properly.
2. Dehydration. In these steps the water in the sample will be substituted by another solution compatible with the resin. In this protocol we use methyl acrylate resin (Technovit 7100; See materials and equipment section) and ethanol solution.
  a. Remove water using a pipette.
  b. Add a volume (use the same volume of fixative used) of 20% ethanol, incubate for 2 h at 4°C.
  c. Remove the solution, add a volume of 40% ethanol, incubate for 2 h at 4°C.
  d. Remove the solution, add a volume of 60% ethanol, incubate for 2 h at 4°C.
  e. Remove the solution, add a volume of 80% ethanol, incubate for 2 h at 4°C.
  f. Remove the solution, add a volume of 100% ethanol, incubate for 2 h at 4°C.

***Note:*** The protocol can be paused at this point. Samples in 100% ethanol can be stored at 4°C covered from light for 1–2 days. Optional: pause in step e) leave in 80% ethanol for 14–17 h at 4°C and continue the next day.

3. Pre-infiltration. This is an intermediate step to treat the sample with the resin.
  a. Prepare the pre-infiltration solution, in this case, ethanol:Technovit basic solution (1:1).
  b. Remove the ethanol solution from the tubes and add the same volume of pre-infiltration solution.
  c. Incubate for 2 h at 22°C–25°C.
4. Infiltration. In this step the sample will be embedded with one of the components of the resin.
  a. Preparation of the infiltration solution in advance.
    i. The Technovit infiltration solution is prepared by combining 100 mL of Technovit basic solution and 1 bag (1 g) of Hardener I and mixing for 3 h with a magnetic stirrer.
    ii. After preparation, the solution must be stored at 4°C and is stable up to 1 month.
  b. Remove the pre-infiltration solution using a pipette and add the same volume of infiltration solution. Incubate for at least 2 h at 22°C–25°C. For better results incubate for 14–17 h at 4°C. See Note.

***Note:*** The protocol can be paused at this point. Samples can stay in infiltration solution for several days at 4°C. The solution stays liquid. The infiltration solution causes the sample to become slightly brittle/breakable.

5. Polymerization. In this step, the sample will be embedded in the resin to make solid blocks.
  a. Take out the sample from the tube and place it in a petri dish.
    i. Use fine tweezers to dissect it and select the region of interest of the sample.
    ii. Depending on the sample size, select the appropriate disposable base mold size (Electron Microscopy Sciences, USA; e.g., 5 × 5, 10 × 10 or 15 × 15 mm; Figure 2A).
      ***Optional:*** unused sample can be stored in the same tube in the same infiltration solution and stored at 4°C for possible future use.
  b. Prepare the polymerization solution.
    i. Mix the Technovit infiltration solution with hardener II (15:1); mix by pipetting.
    ii. The polymerization solution can be used up to 15 min after preparation. See Note.
      ***Note:*** To avoid wasting solution, only prepare the amount you will need for the following step. After 15 min the solution will solidify, thus not usable anymore.
  c. Add some polymerization solution to the base mold. For the small mold bases, you can use 200 μL (Figure 2B1). For this step, you only need to cover the sample, not the entire base mold.
    i. Place the sample in the base mold and try to position it in the desired position. The sections will be parallel to the bottom of the mold. You can use tweezers or needles to help. In the beginning the solution has not much viscosity yet and the tissue can move a bit.
    ii. Wait for the solution to harden, it will take around 15 min for the solution to get viscous, to check this process you can use a needle to pinch inside the mold base and check if it is getting hard. Additionally, you can use the rest of the infiltration solution to monitor the process.
    iii. Once the solution is hard enough to keep the sample in its place (to check this try to move the sample with the needle) proceed to the next step.
  d. Place an embedding cassette over the base mold with the sample and add more polymerization solution to fill the base mold (for small base molds, you can use around 3 mL) (Figure 2C1).
    ***Note:*** In this way, the block with the sample will now be attached to the embedding cassette, which will serve as support for the block with sample, and is needed to mount the block on the microtome (but the support needed depends on the microtome used). Wait at least one hour before moving the mold. Alternatively, you can use Technovit 3040 (is yellow) to fill the base mold to attach it to the embedding cassette, which is a yellow (Figure 2C2).
  e. Let the samples polymerize for 14–17 h at 28°C–37°C.
    ***Note:*** Tissues must have enough space during the fixation process, we recommend a 10:1 fixative:tissue in volume. If there is too much tissue squeezed into a tube, the fixation will be less efficient; air bubbles stay trapped in between the tissue. Furthermore, tissue can also be trapped in the tube and no sinking of the tissue can be observed.
    **CRITICAL:** Handle Technovit 3040 when liquid in the fume hood to avoid inhaling toxic vapors.
    **CRITICAL:** Be sure that the base mold and embedding cassette are compatible with the microtome.

## Key resources table

**Table:** 

REAGENT or RESOURCE	SOURCE	IDENTIFIER
**Antibodies**		

1ab: Rabbit anti-trans-zeatin riboside (1:500)	OlChemIm, Czech Republic	Cat#004 0312
1ab: Rabbit anti-indole-3-acetic-acid (N1) (1:500)	OlChemIm, Czech Republic	Cat#004 1536
Optional 1ab: rabbit anti-trans-zeatin riboside (1:500)	Agrisera, Sweden	Cat#AS09 414
Optional 1ab: rabbit anti-indole-3-acetic-acid (N) (1:500)	Agrisera, Sweden	Cat#AS09 421
1ab: Rat heteromannan monoclonal antibody LM21 (1:500)	Kerafast, USA (previously by PlantProbes, UK)	Cat#ELD019
2ab: Goat anti-rabbit IgG (H&L), DyLight 488 conjugated (1:2000)	Agrisera, Sweden	Cat#AS09 633
2ab: Goat anti-rat IgM mu chain, DyLight 488 conjugated (1:2000)	Abcam, UK	Cat#ab98368
2ab: Goat anti-rabbit IgG antibody, alkaline phosphatase conjugate (1:2000)	Merck, USA	Cat#AP132A

**Chemicals, peptides, and recombinant proteins**		

Technovit 7100 (resin)	Heraeus Kulzer, Germany (alternative: Electron Microscopy Sciences, USA)	https://kulzer-technik.com (alternative: EMS Cat#14653)
Technovit 3040 (glue)	Heraeus Kulzer (alternative: Electron Microscopy Sciences, USA)	https://kulzer-technik.com (alternative: EMS Cat#14653)
NBT-BCIP solution	Merck, USA	Cat#72091
Bovine serum albumin, fraction V	GoldBio.com, USA	CAS 9048-46-8, Cat#A-420-100

**Other**		

Vacuum pump	Gast Manufacturing, USA	DOA-P704-AA
Desiccator	NA	NA
Microtome	Leica, Germany	NA
Fume hood	NA	NA
Pipettes	NA	NA
Bright-field, fluorescence, or confocal microscope	Zeiss, Germany	NA
Falcon tubes 15 mL, 50 mL	NA	NA
Tissue culture 24-well plates	NA	NA
Tweezers	NA	NA
Plastic molds – disposable base mold, different sizes	Electron Microscopy Sciences, USA	Cat#70915
Tissue-Tek process/embedding cassettes without lid	Electron Microscopy Sciences, USA	Cat#62520-W
Microscope slides and cover slips	NA	NA
Fluoroshield, histology mounting medium	Sigma, USA	Cat#F6182
Cytoseal 60 mounting medium	Electron Microscopy Sciences, USA	Cat#18007

1ab: primary antibody; 2ab: secondary antibody.

**Antibody alternatives**: Antibodies can be obtained from different suppliers.

OlChemIm, Czech Republic: https://www.olchemim.cz/

Agrisera, Sweden: https://www.agrisera.com/

Kerafast, USA: https://www.kerafast.com/

Abcam, UK: https://www.abcam.com/

Merck, USA: https://www.merckmillipore.com/

## Materials and equipment

**Table:** Fixation buffer

Reagent	Amount	Final concentration
Paraformaldehyde	4.5 mL of 10% stock	3%
Microtubule-stabilizing buffer	7.5 mL 2× MTSB	1× MTSB
Glutaraldehyde 50% solution	150 μL	0.5%
10% Triton X-100	150 μL	0.1%
Milli-Q water	Adjust to 15 mL

***Note:*** Prepare in the fume hood to avoid inhaling formaldehyde vapors.

***Note:*** Prepare paraformaldehyde stock solution: Dissolve 2 g of paraformaldehyde powder in 20 mL of hot Milli-Q water for a 10% stock solution. Add 1–2 drops of 1 M KOH solution for faster dilution. Use directly or this stock solution can be stored in 5 mL aliquots at −20°C and use them within 2 weeks. Prepare in the fume hood to avoid inhaling formaldehyde vapors.

***Note:*** 10% Triton X-100 means commercially available Triton X-100 diluted 10-fold.

**CRITICAL:** Fixation buffer is toxic due to paraformaldehyde.

**Table:** Microtubule-stabilizing buffer; 2× MTSB^7^

Reagent	Amount	Final concentration
PIPES	15 g	100 mM
EGTA	1.90 g	10 mM
MgSO_4_·7H_2_O	1.22 g	10 mM
KOH	2.5 g	100 mM

Dissolve in 500 mL distilled water and adjust pH to 7.0 with 10 M KOH solution. This buffer can be stored at 22°C–25°C for six months.

**Table:** Phosphate buffered saline; 10× PBS

Reagent	Amount	Final concentration
NaCl	80 g	1.3 M
KCl	2 g	27 mM
Na_2_HPO_4_	14.4 g	100 mM
KH_2_PO_4_	2.4 g	18 mM

Dissolve in 800 mL distilled water and adjust pH to 7.4 with 1 M HCl, adjust to 1 L, autoclave at 121°C for 15 min. This buffer can be stored at 22°C–25°C for six months.

**Table:** Tris-buffered saline; 10× TBS

Reagent	Amount	Final concentration
Tris	60.6 g	0.5 M
NaCl	87.6 g	1.5 M

Dissolve in 800 mL distilled water and adjust pH to 7.5 with 1 M HCl, adjust to 1 L. This buffer can be stored at 22°C–25°C for six months.

• Absolute ethanol.
• Technovit 7100 (Heraeus Kulzer). Technovit 7100 Combipack contains: 1 × 500 mL basic solution, 5 × 1 g bags of hardener 1, 1 × 40 mL hardener 2. (https://kulzer-technik.com/en-kt/en-kt/products/technovit-7100.html) Alternative: available at Electron Microscopy Sciences, USA: Catalog #14653.
• Infiltration solution: 100 mL of Technovit 7100 basic solution and 1 g (1 bag) of Technovit 7100 hardener I. Dissolve the powder, takes at least 3 h.

***Note:*** Store in a closed bottle at 4°C. Stable up to 4 weeks.

• Polymerization solution: Mix infiltration solution with Technovit 7100 hardener II in a 15:1 ratio. Mix for 3 min by pipetting. It starts polymerizing in 5 min.
• Technovit 3040 preparation: Mix powder and liquid components in a 2:1 ratio in a fume hood as follows: Put the liquid component in a disposable 50 mL falcon tube. Add half of the powder component. Cap and mix vigorously. Add remaining of the powder component. Mix for 40 s. The mix starts polymerizing after 30 s and should be poured quickly. It will solidify within 5 min. This powder is not included in the Technovit 7100 Combipack.

***Note:*** Prepare in the fume hood to avoid inhaling formaldehyde vapors.

• Tween 20.
• Blocking solution: 1% albumin fraction V from BSA in 1× MTSB (for fluorescent antibodies).
• Blocking solution: 1% albumin fraction V from BSA in 1× PBS (for non-fluorescent antibodies).
• Primary antibody (1ab) solution: Dilute primary antibodies in blocking solution before using.

Primary antibody concentration should be determined experimentally and can vary between 1:20 and 1:1000 since it is heavily dependent on epitope concentration.

• Secondary antibody (2ab) solution: Secondary antibody is diluted in a range from 1:200 to 1:2000 in blocking solution right before use. If provided, follow manufacturer’s instructions.

***Note:*** Prepare only the amount needed per experiment, usually 500 μL per sample. Do not store diluted antibody solutions.

• Detection buffer for NBT-BCIP: 100 mM Tris-HCl pH 9.5, 100 mM NaCl, 50 mM MgCl_2_. This buffer can be stored at 22°C–25°C for six months.
• TE: 100 mM Tris-HCl pH 8, 50 mM EDTA pH 8. This buffer can be stored at 22°C–25°C for six months.

## Step-by-step method details

### Section preparations

**Timing: 2–4 h**

In this step, you will generate transverse or longitudinal sections of your sample to use for the immunolabeling procedure.

1. Block trimming.
  a. After polymerization, the resin-embedded sample inside the block should be trimmed to remove excess resin regions and to orient the sample in the block for microtome sectioning (Figure 2D).
    ***Note:*** The goal of this step is to help obtain the tissue sections easily by removing excess resin regions without sample from the block. The width of the block at this point will define the section size, and the number of sections that can be handled during the experiments. Depending on the sample, an optimal section size is 3 × 3 mm to 5 × 5 mm.
  b. Use a blade or scalpel to remove resin without sample, be sure to keep a square shape at the end (Figure 2D). Keeping a square shape makes it easier to section.
2. Sectioning.
  a. Make the basic adjustments to the microtome (knife angle, sample position, thickness selection), be sure that everything is correctly set and place the base mold with the sample to start the cutting process.
  b. Obtain sections. Sections with a thickness of 10–16 μm are recommended.
    ***Note:*** The first sections you obtain could not have sample in it (Tissue is not directly at the surface of the block, first some sections must be cut that will only have plastic resin). Check the presence of the correct sample within the section by collecting some sections and observing them under a stereo microscope. Once you start getting sections with the sample proceed to the next step.
  c. Grab the sections with some tweezers and without releasing them, dip them for a few seconds in a well containing distilled water, this step will eliminate any debris. The well used for this step can be in another box.
  d. After the washing step, transfer up to 5–10 sections to 1 well of a 24-well plate containing 1 mL of Milli-Q water (per well), to continue with the protocol.
    ***Note:*** Include samples for the negative and positive controls (Figure 2).
3. Blocking
  a. Prepare blocking solution (see the materials and equipment Section).
  b. Remove water from the well with a pipette and add blocking solution. The working volume will depend on the number of sections you have in the well, usually 1 mL is enough for up to 5–10 sections.
  c. Wash three times with blocking solution 10 min each.

**CRITICAL:** To avoid the sections sticking together in the wells, try to use small sections, less than 5 × 5 mm. If the sample is too large or complex, try to dissect it first and embed it in separate blocks. Sections should not overlap in the solution, otherwise they will stick together.

### Immunolabeling using fluorescent antibodies

#### Incubation with primary antibody

**Timing: 14–17 h**

During this step, primary antibodies bind to their epitope molecules in the sample.

4. After Step 3c, remove the blocking solution from the wells using a pipette.
5. Replace blocking solution with 500 μL of primary antibody solution (diluted).
  a. Previously prepare the primary antibody (from 1:20 to 1:1000; see Note) in blocking solution supplemented with 0.025% Tween 20.
  b. Do not agitate samples during incubation with primary antibodies.
  c. Always include control samples that were not incubated with primary antibody. In this case, carry incubation in blocking solution with 0.025% Tween 20.

***Note:*** Another possible control is adding a standard solution of the epitope to one of the samples being incubated with primary antibody. Because of competitive reaction, this sample should have lower signal intensity but same spatial localization.

6. Incubate for 14–17 h at 4°C; place the lid on the plate and protect from light. No agitation is needed.
7. After incubation, wash 2 times with 1 mL 1× MTSB solution,^7^ 5 min each.

***Note:*** Primary antibody concentrations vary. A guideline is to follow the manufacturer’s recommendation. In our hands, we use a 1:500 dilution for the hormone antibodies (OlChemIm).

Cytokinin: anti-trans-zeatin stock 4.3 μg/μL; 1:500 dilution means 0.0086 μg/μL or 8.6 μg/mL.

Auxin: anti-indole-3-acetic-acid stock 3.12 μg/μL; 1:500 dilution means 0.00624 μg/μL or 6.24 μg/mL.

When using the approach as the example in Figure 2, if 4 wells are used for one experiment; first well is the negative control (NC) without antibody, for the next 3 wells prepare 1.5 mL blocking solution with 0.025% Tween 20 + 3 μL antibody of the stock (gives a 1:500 dilution) and then use 0.5 mL per well.

#### Incubation with secondary antibody

**Timing: 2 h**

Secondary antibodies can be conjugated to a reporter such as a fluorescent molecule. The secondary antibody binds to the primary antibody, which is generated in a different species; for example, a goat generated anti-rabbit secondary antibody that will bind to a primary antibody produced in rabbit.

8. Remove the washing 1× MTSB solution.
9. Blocking: add 1 mL of 1× MTSB + 1% BSA, incubate for 5 min. Repeat 3 times.
10. Add 500 μL of the secondary antibody solution previously prepared in blocking buffer (1:200 to 1:2000 dilution; depending on the antibody concentration; see Note).
11. Incubate for 1–2 h at 22°C–25°C, place the lid on the plate, in the dark.
12. Wash 3 times with 1 mL 1× MTSB after completing, 5 min each.
13. Proceed to sample mounting and observation (see sample mounting and observation section, Step 29).

***Note:*** Secondary antibody concentrations vary. A guideline is to follow the manufacturer’s recommendation. In our hands, we use a 1:1000 dilution for the Dylight 488 antibody (Abcam). The stock is 0.5 μg/μL; a 1:1000 dilution means 0.5 μg/mL.

When using the approach as the example in Figure 2, if 4 wells are used for one experiment; prepare 2 mL of 1× MTSB with 1% BSA + 2 μL antibody of the stock (gives a 1:1000 dilution) and then use 0.5 mL per well.

***Note:*** With the use of a secondary antibody conjugated with Alkaline Phosphatase, we obtained better results incubating it for at least 3 h or longer. With the use of a secondary antibody conjugated with a fluorescent molecule, e.g., DyLight 488, a 1–2 h incubation period is sufficient.

### Immunolabeling using non-fluorescent antibodies

#### Incubation with primary antibody

**Timing: 14–17 h**

During this step, primary antibodies bind to their epitope molecules in the sample.

14. After Step 3c, remove the blocking solution from the wells using a pipette.
15. Replace blocking solution with 500 μL of primary antibody solution (diluted).
  a. Previously prepare the primary antibody (from 1:20 to 1:1000; see Note) in blocking solution (1× PBS + 1% BSA) supplemented with 0.025% Tween 20.
  b. Do not agitate samples during incubation with primary antibodies.
  c. Always include control samples that were not incubated with primary antibody. In this case, carry incubation in blocking solution with 0.025% Tween 20.

***Note:*** Another possible control is adding a standard solution of the epitope to one of the samples being incubated with primary antibody. Because of competitive reaction, this sample should have lower signal intensity but same spatial localization.

16. Incubate for 14–17 h at 4°C; place the lid on the plate. No agitation is needed.
17. After incubation, wash 2 times with 1 mL 1× PBS solution, 5 min each.

***Note:*** Primary antibody concentrations vary. A guideline is to follow the manufacturer’s recommendation. In our hands, we use a 1:500 dilution for the hormone antibodies (OlChemIm).

Cytokinin: anti-trans-zeatin stock 4.3 μg/μL; 1:500 dilution means 0.0086 μg/μL or 8.6 μg/mL.

Auxin: anti-indole-3-acetic-acid stock 3.12 μg/μL; 1:500 dilution means 0.00624 μg/μL or 6.24 μg/mL.

When using the approach as the example in Figure 2, if 4 wells are used for one experiment; first well is the negative control (NC) without antibody, for the next 3 wells prepare 1.5 mL blocking solution with 0.025% Tween 20 + 3 μL antibody of the stock (gives a 1:500 dilution) and then use 0.5 mL per well.

#### Incubation with secondary antibody

**Timing: 5 h**

Secondary antibodies can contain a conjugated enzyme that generates a visible colored product. The secondary antibody binds to the primary antibody, which is generated in a different species; for example, a goat generated anti-rabbit secondary antibody that will bind to a primary antibody produced in rabbit.

18. Remove the washing 1 mL 1× PBS solution.
19. Blocking: add 1 mL of 1× TBS + 1% BSA, incubate for 5 min. Repeat 3 times.
20. Add 500 μL of the secondary antibody solution previously prepared in blocking buffer (1:200 to 1:2000; depending on the antibody concentration; see Note).
21. Incubate for 3–5 h at 22°C–25°C, place the lid on the plate, in the dark.
22. Wash 3 times with 1 mL 1× TBS, 5 min each, at 22°C–25°C.
23. Proceed to Staining/revealing reaction Section (see staining/revealing reaction section, Step 24).

***Note:*** Secondary antibody concentrations vary. A guideline is to follow the manufacturer’s recommendation. In our hands, we use a 1:2000 dilution for the anti-rabbit IgG antibody, Alkaline Phosphatase conjugate (Merck). The stock is 1.6 mg/mL; a 1:2000 dilution means 0.0008 mg/mL or 0.8 μg/mL.

When using the approach as the example in Figure 2, if 4 wells are used for one experiment; prepare 2 mL of TBS with 1% BSA + 1 μL antibody of the stock (gives a 1:2000 dilution) and then use 0.5 mL per well.

***Note:*** With the use of a secondary antibody conjugated with Alkaline Phosphatase, we obtained better results incubating it for at least 3 h or longer. With the use of a secondary antibody conjugated with a fluorescent molecule, e.g., DyLight 488, a 1–2 h incubation period is sufficient.

***Note:*** Without systematic testing, we have the idea that the results are similar using either MTSB or TBS buffer independently whether fluorescent or non-fluorescent antibodies are used. PBS and TBS buffers are slightly easier to prepare. Important, when using an Alkaline Phosphatase conjugated antibody, do not use PBS because it interferes with the signal, but use TBS. Important, to observe hormones by immunolocalization, the MTSB buffer must always be used in the fixation solution (see sample embedding Section).

### Staining/revealing reaction (for non-fluorescent secondary antibodies)

**Timing: 10 h**

When the secondary antibody is conjugated with alkaline phosphatase (AP), the substrate NBT-BCIP solution is used, and the enzyme reaction will produce a purple signal (Figure 3).

24. After Step 23, remove the 1× TBS solution.
25. Wash 3 times with blocking solution (1× TBS + 1% BSA), 5 min each.
  a. Mix Nitro blue tetrazolium chloride (NBT) and 5-bromo-4-chloro-4-indolyl phosphate (BCIP), check the manufacturer’s recommendation. See note below.
  b. Prepare the substrate solution: detection solution + NBT-BCIP solution.
  c. Mix Nitro blue Tetrazolium Chloride (NBT) and 5-Bromo-4-chloro-4-indolyl phosphate (BCIP), check the manufacturer’s recommendation. See note below.
  d. Add 1 mL solution to each well and incubate for 3–5 h in the darkness at 22°C–25°C.
26. Stop the reaction by adding 1 mL TE solution to each well, leave for 10 min.
27. Wash 3 times with Milli-Q water, 5 min each.
28. Proceed to sample mounting and observation (see sample mounting and observation section, Step 31).

***Note:*** Stock solution NBT-BCIP contains 18.8 mg/mL NBT and 9.4 mg/mL BCIP (Merck). The final concentration needed for the reaction is 0.15 mg/mL NBT and 0.075 mg/mL BCIP.^8^ When using the approach as the example in Figure 2, if 4 wells are used for one experiment, this means that mix 8 mL detection buffer + 64 μL of NBT-BCIP stock solution and then use 1 mL of this mixture per well.

### Sample mounting and observation

**Timing: 3 h or 19 h**

Mount samples on a microscope glass slide to observe the result of the immunolabeling. Depending on the type of secondary antibody used, there are two ways to observe the result:

For fluorescent conjugated labels.

29. After Step 13, use tweezers to transfer the samples from the well plate onto a microscope slide and place a cover slip.

***Note:*** Try to grab the sections from one corner. Expand and organize for viewing. Placing a drop of antifade fluorescent mounting medium (Fluoroshield; see materials and equipment Section) is helpful both to mount the sections and to preserve fluorescence. Add a cover slip. Alternatively, add 50% glycerol and add a cover slip.

30. Capture images using a confocal or fluorescence microscope.

For non-fluorescent conjugated labels.

31. After Step 28, place some glass slides on a slide warmer at 42°C and add some distilled water on each slide.
32. Use tweezers to transfer the sections to the glass slide on the water, be sure the sections are fully expanded, you can use some tweezers to help.
33. Let the water evaporate.
34. Cover the slides with mounting medium (Cytoseal 60) and wait for 16 h (at 22°C–25°C) to let the mounting medium dry and generate permanent slides. Alternatively, add 50% glycerol and add a cover slip, and observation can be done directly.
35. Slides are ready to observe under a light microscope.

***Note:*** Slides with non-fluorescent samples can be observed later (up to days/week). Fluorescent samples should be observed immediately (the same day).

## Expected outcomes

The expected outcomes are to detect the distribution of metabolites or e.g., cell wall components in resin embedded samples. Depending on the secondary antibody used, the signal can be fluorescent or non-fluorescent. In Figure 3, different immunolabeling signals are shown. The first is the immunolabeling of mannan polysaccharides in a mature Arabidopsis gynoecium, and changes in distribution during medial region development in wild-type samples, or changes observed in mutants.^1^^,^^2^ Second, we have also used this protocol to study the distribution of cytokinin during gynoecium development in Arabidopsis, distribution of cytokinin and auxin in ovules in Arabidopsis (unpublished; Figure 3), and the distribution of cytokinin and auxin during maize regeneration.^3^ Variations on this protocol can be used, for instance for whole mount immunolabeling.^4^^,^^5^

## Limitations

The limitation of this protocol is the availability of a suitable antibody. Commercial antibodies can be purchased from different suppliers. In some countries, it can be difficult and time consuming to buy and receive antibodies. Custom antibody production might be an option. Another limitation might be insufficient tissue fixation, which can result in bad sections or separation of samples from the embedded resin during the washing steps.

## Troubleshooting

### Problem 1

Loss of sections during manipulation.

### Potential solution

• Try not to use sections with sizes that are too small, this can be adjusted at the beginning during the block trimming (see section preparations section, Step 1).

### Problem 2

Excess of signal.

### Potential solutions

• When using non-fluorescent antibodies, after 3.5 h in detection buffer with NBT-BCIP, you can check the staining reaction, depending on the signal intensity you can stop or leave it longer (see staining/revealing reaction section, Step 25d).
• Use a lower concentration of primary antibody.

### Problem 3

Lack of signal.

### Potential solutions

• The number of washing steps can be reduced, for instance, reduce to a single wash with Milli-Q water (instead of 3 washes) in the last wash step, before mounting (see staining/revealing reaction section, Step 27).
• Increase antibody concentration.
• Verify that no additional treatment is necessary to make the molecule of interest available for antibody binding (e.g., mannans in the cell wall; before immunolabeling, a treatment of the sections with 1 M KOH for 1 h is needed to expose hidden mannans^2^^,^^6^). This treatment can be done on the sections in a 24-well plate after Step 2d (see section preparations section), afterwards continue with Step 3 (see section preparations section).^2^ There is no special treatment necessary to detect hormones.
• Verify that a compatible secondary antibody is used to recognize primary antibody.
• Consider using a new batch of antibody (primary or secondary).

### Problem 4

Sections in the wrong position.

### Potential solution

• The sample inclusion in the resin is critical for the protocol (Refer to sample embedding section, Step 5); a sample in the correct position will provide good sections. It is recommended to dissect the sample before embedding to get only the region of interest to be included in the resin; for placing the sample, we suggest doing some transverse cuts at the base of the sample to help to place the sample in a stand position.
• For some samples, it is recommended to do a two-step resin inclusion; the first step will generate only a resin block with the sample, which can then be trimmed to generate a cube with the sample in the right angle, which can then be glued in the correct orientation on the embedding cassette.

### Problem 5

Antibody storage.

### Potential solution

• To avoid antibody degradation due to the frequent freezing/unfreezing, we recommend preparing single-use aliquots with 5 or 10 μL of the antibody. Long time storage at −20°C; short term storage at 4°C is fine.

## Resource availability

### Lead contact

Further information and requests for resources and reagents should be directed to and will be fulfilled by the lead contact, Stefan de Folter (stefan.defolter@cinvestav.mx).

### Materials availability

This study did not generate new unique reagents.

### Data and code availability

This study did not generate new data or code.
